# Lymphangioma Circumscriptum of the Vulva: A Case Report

**DOI:** 10.7759/cureus.106294

**Published:** 2026-04-01

**Authors:** Ikran A Haji Adan Mohamud

**Affiliations:** 1 Obstetrics and Gynaecology, Princess Royal University Hospital, Orpington, GBR

**Keywords:** case report, dermatological condition, gynecology, lymphangioma circumscriptum, surgical excision, vulvar lesions, vulvar swelling, vulvectomy

## Abstract

Vulvar lymphangioma circumscriptum is a rare, benign lymphatic malformation that can mimic infectious or neoplastic lesions, often leading to delayed diagnoses. This report presents the case of a 48-year-old woman who presented with a 10-year history of wart-like lesions on the vulva, causing soreness, pruritus, and an impaired quality of life. Examination revealed multiple fluid-filled papules and swelling of the left labia majora. She underwent surgical excision, and histopathology confirmed lymphangioma circumscriptum without evidence of malignancy. At 12-month follow-up, she remained asymptomatic with no recurrence. This case highlights the importance of considering lymphangioma circumscriptum in chronic vulvar lesions and demonstrates that surgical excision can provide effective symptom relief.

## Introduction

Lymphangioma circumscriptum vulva is a rare, benign skin condition characterized by clusters of small, fluid-filled sacs (vesicles) on the vulva that can resemble genital warts. It is caused by a localized defect in the lymphatic system, which can be congenital or acquired due to factors like treatment history for tuberculosis, pelvic surgery, and radiation therapy, often linked to conditions like cervical cancer [1]. Symptoms include itching, burning, and sometimes oozing of fluid, and a biopsy is required for a definitive diagnosis [2]. 

This report presents the case of a 48-year-old para 3 woman who presented with wart-like lesions affecting the vulva. She was diagnosed with acquired lymphangioma circumscriptum of unknown cause and was treated with a superficial vulvectomy. Over 12 months of follow-up, she remained asymptomatic with no evidence of recurrence.

## Case presentation

A 48-year-old para 3 woman presented to the gynecology outpatient department with a decade-long history of wart-like lesions affecting the vulva. The lesions had gradually increased in size and were accompanied by soreness, itching, and a significant reduction in her quality of life. She additionally complained of lower abdominal discomfort, back pain, and intermittent leg swelling. Prior treatments, including antibiotics, antifungals, and antiviral medications, had not provided any improvement. Her obstetric history included uncomplicated vaginal deliveries, and she had no notable past medical or surgical history.

**Figure 1:**
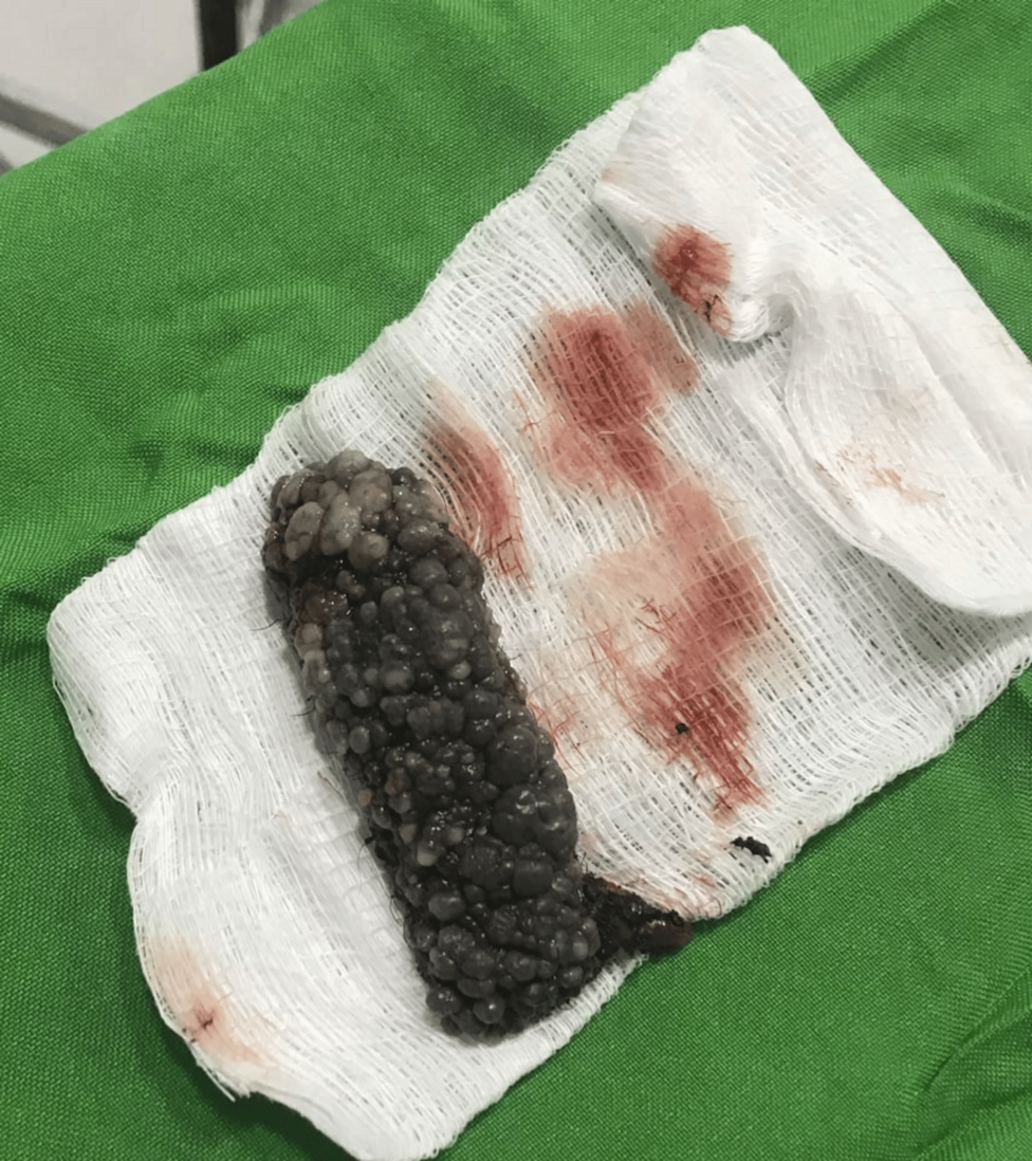
Gross morphology of lymphangioma circumscriptum of the vulva

On examination, there was a pronounced swelling of the left labia majora with clusters of papules and vesicles, some displaying a warty appearance and exuding fluid (Figure 1). The general assessment was unremarkable. Her routine investigations were within normal limits, and pelvic ultrasound did not reveal any abnormalities. After assessment in the gynecology department, she was counselled about the available treatment options and the risk of recurrence. With written informed consent, she underwent an elective procedure consisting of unilateral superficial vulvectomy with primary closure of the skin, performed under spinal anesthesia.

**Figure 2:**
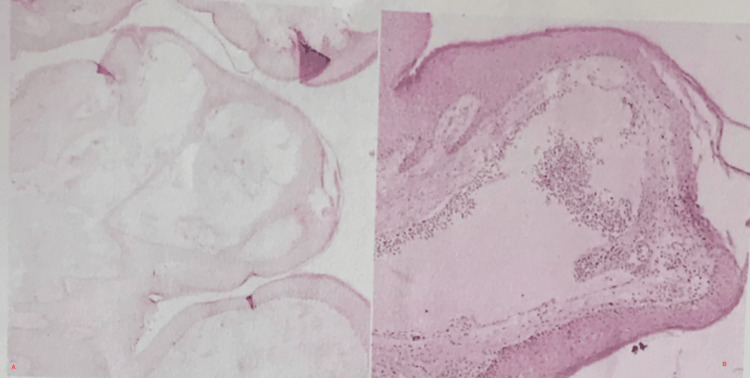
Microscopically, there were expanded lymphatic spaces within the superficial dermis, accompanied by a mild inflammatory response (A). The epidermis above these areas showed patches of surface thickening and zones where the stratum spinosum layer appeared reduced (B). There was no evidence of malignancy.

The excised tissue was submitted for histopathological analysis, which demonstrated multiple dilated vascular channels within the papillary dermis, containing fibrin and occasional erythrocytes, lined by a single layer of endothelial cells (Figure 2). These findings confirmed the diagnosis of vulvar lymphangioma circumscriptum. At 12-month follow-up, the patient remained symptom-free with no evidence of recurrence.

## Discussion

Our case of vulvar lymphangioma circumscriptum aligns with the clinical patterns described in prior reports but also expands on the existing literature in meaningful ways. Previous reviews and case series demonstrate the rarity of vulvar lymphangioma circumscriptum and highlight how often it is misdiagnosed as genital warts or other dermatological conditions due to its nonspecific clinical appearance. For example, a comprehensive review found that typical presentations include persistent clusters of translucent vesicles on the vulva, frequently prompting misdiagnosis and delayed treatment [3, 4]. 

There is currently no universally accepted treatment approach for vulvar lymphangioma circumscriptum, and management is tailored to the individual patient based on symptoms, lesion extent, and available therapeutic options. Treatment options range from conservative approaches such as decongestive physiotherapy, including manual lymph drainage, exercise, and compression, to more invasive methods such as abrasive therapy, sclerotherapy, electrocoagulation, carbon dioxide laser therapy, and surgical excision. Although lesion recurrence is common, surgical excision is often preferred. The reported recurrence rate following surgical management is approximately 23.1% over a follow-up period of six to 81 months, while recurrence may be up to twice as high in patients managed without surgery. Recurrence after radical excision is higher when the initial lesion exceeds 7 cm in diameter compared with smaller lesions treated by local excision [4]. In our case, superficial vulvectomy was chosen due to the extensive nature of the lesion and the lack of alternative treatment options. The patient was symptomatic, making conservative management inappropriate.

Surgical excision has been a common treatment modality in such cases. In a case series involving three older women (ages 45-60), local surgical excision was the preferred approach, and no recurrence was reported over follow-up [4]. Our findings are consistent with this. In our patient, surgical removal resulted in substantial symptomatic relief without recurrence at the 12-month follow-up, supporting excision as a viable option.

In summary, our case reinforces the importance of considering lymphangioma circumscriptum in the differential diagnosis of persistent vulvar lesions, supports the effectiveness of surgical excision (even for extensive disease), and contributes to the limited body of literature by reporting a stable, recurrence-free outcome in a patient without identifiable predisposing factors.

## Conclusions

Lymphangioma circumscriptum should be considered in patients presenting with persistent vulvar papular or vesicular lesions that do not respond to standard treatments, as timely histological confirmation is essential for accurate diagnosis and appropriate management. Early recognition and accurate diagnosis, often supported by histopathology, are essential to guide appropriate management and prevent unnecessary treatments. While therapeutic options, including surgical excision, laser therapy, and other local treatments, may improve symptoms, recurrence is not uncommon. Greater clinical awareness of this rare condition is crucial to ensure timely diagnosis, appropriate management, and improved patient outcomes.
